# Possible unusual presentation of opioid side effect in a child: a case report

**DOI:** 10.1186/s12887-023-04499-9

**Published:** 2024-01-15

**Authors:** Moukhaiber Jihane, Lakissian Zavi, Majdalani Marianne

**Affiliations:** 1 Department of Pediatrics and Adolescent Medicine, American University of Beirut Medical Center, Beirut, Lebanon; 2 Faculty of Medicine, American University of Beirut Medical Center, Beirut, Lebanon

**Keywords:** Opioid, Delirium, Hysteria, Naloxone

## Abstract

Atypical presentations are commonly encountered in the Pediatric intensive care unit (PICU) but having a high index of suspicion is crucial to prevent or treat severe and life-threatening conditions. This case describes the clinical presentation and course of a 14-month-old girl with congenital heart disease who was admitted to the PICU after cardiac repair and remained agitated, irritable, in hysteria and delirium despite adequate sedation. Different measures to relieve her condition were attempted but to no avail. All the common causes of this atypical presentation including pain, ventilator induced agitation, low cardiac output syndrome (LCOS), opioid side effects, toxicity, opioid induced neurotoxicity (OIN) as well as withdrawal syndrome were ruled out. However, the use of naloxone as a last resort after exhausting all the other options has led to immediate and successful reversal of her symptoms.

## Introduction

Opioids are commonly used in the Pediatric Intensive Care Unit (PICU) especially during the postoperative period as analgesics and sedatives. Despite having well known side effects [1], in some multifactorial instances [2, 3], opioid metabolites can accumulate in the body leading to overdose, opioid induced neurotoxicity (OIN) or opioid withdrawal syndrome if improperly discontinued. While the constellation of symptoms for each complication might not all be present, its treatment or reversal is unique for each. We present the case of a 14-month-old girl who experienced severe hysteria and irritability after opioid use post operatively, findings that are similar to those seen in OIN, but who failed to respond to all known therapeutic measures and was fully reversed after the administration of naloxone.

## Case presentation

Our patient is an 8.2 kg, 14-month-old girl with a ventricular septal defect (VSD) status post main pulmonary artery (MPA) banding, who was admitted to our hospital for VSD closure and MPA de-banding. Her operation was uncomplicated, and she was transferred to the PICU for post-operative management while intubated and sedated.

On post-op day 1, she had transient low cardiac output syndrome (LCOS), requiring inotropic and vasopressor support with milrinone, epinephrine and norepinephrine, and she had mild transient acute kidney injury (AKI) (creatinine increased from 0.3 to 0.6 mg/dl) without uremia. Her AKI and LCOS resolved on post operative day 2, and her inotropes weaned off on post operative day 3.

The patient’s initial sedation consisted of morphine (0.02 mg/kg/hour) and midazolam (0.05 mg/kg/hour) infusions, but since presentation, she was severely agitated, requiring multiple boluses of midazolam and morphine, as well as sedatives adjustment, first by gradual increase in the infusion rates reaching midazolam (0.2 mg/kg/hour) and morphine (0.1 mg/kg/hour), then by adding dexmedetomidine drip reaching gradually (0.4 mcg/kg/hour), using ketamine pushes as well, but to no avail. Decision taken to switch morphine to fentanyl drip (1 mcg/kg/hour), but no change in her status was noted. On post operative day 3, she was hemodynamically stable, the decision was to extubate her on Dexmedetomidine only, hoping to relieve her agitation as well. She was successfully extubated, but she continued to be inconsolable, agitated, hysteric and incapable of fixating nor tracking, a situation that distraught her parents so badly.

Pain was ruled out as pain killers and a fentanyl push did not relieve them. Additionally, low cardiac output was ruled out, as the patient was hemodynamically stable with good urine output, warm extremities and normal laboratory tests including serial lactate levels. Withdrawal was ruled out, as same symptoms were present since admission to PICU, she was maintained on opioids and midazolam for a short period of 3 days, and her symptoms did not resolve after low doses of fentanyl and midazolam pushes. Opioid toxicity was ruled out as she had normal size pupils on exam, no respiratory depression or change in her level of consciousness, no nausea or vomiting, and again these symptoms were present since post operative day 1. Opioid induced neurotoxicity syndrome was also ruled out, since symptoms were neither relieved after switching morphine to fentanyl, nor after stopping all the opioids nor after adding dexmedetomidine, and maintaining good fluid balance.

Prior to performing a computed tomography (CT) scan to rule out any bleeding post bypass surgery, she was given 0.1 mg/kg naloxone dose as a trial; after which she calmed down immediately, and was able to fixate her eyes, look at her mom, recognize her, point toward her milk, drink a little and slept. She woke up calmly the following morning with no signs of agitation. She could fixate, track and interact with her surroundings. The CT scan was within normal limits. She was followed closely and was back to her normal baseline within 2 days.

## Discussion

Our patient underwent cardiac surgery, after which she was maintained on the usual opioid regimen for sedation and analgesia post operatively, but her severe agitation, irritability and hysteria were pertinent since admission to the PICU. Our possible differential diagnosis were pain or suboptimal sedation and analgesia, ventilator induced agitation, LCOS, opioid side effects, opioid overdose/intoxication, OIN, withdrawal syndrome and intracranial bleeding post bypass surgery. Although her symptoms shared some similarities with OIN, they did not fully fit any of the previously mentioned differential diagnosis. The serial physical examinations, the measures and management taken, helped us excluding the possible diagnosis progressively. Finally, the complete and immediate reversal of her symptoms after naloxone administration, pointed toward a possible unusual opioid side effect. To better understand opioid related signs and symptoms, we can divide them to: opioid side effects, overdose or intoxication, OIN and withdrawal syndrome. Please refer to (Table 1) to differentiate between each category and its specific treatment. The metabolism of morphine might be influenced by cardiac surgeries due to their effect on hepatic and renal blood flow, leading to delayed clearance and accumulation of metabolites [2]. Dagan et al. [3] found that clearance was prolonged in children who required inotropic support as well. Our patient underwent cardiac surgery, required inotropic support for 3 days and suffered a transient AKI, all of which may have contributed to an altered metabolism of opioids. Although unusual agitation and delirium are not sure to be a side effect of opioid or an association but it still needs to be considered. In conclusion, this case highlights a possible atypical side-effect of opioids, thus the need to bring awareness about these differential diagnoses in children receiving opioids and not following an expected course during their hospital stay.

**Table 1:** Differential diagnosis and treatment of different opioid related signs and symptoms

	Pathophysiology	Signs and symptoms	Treatment
Opioid side effects [4]	Activation of MOP, DOP and KOP receptors	Sedation, dizziness, pruritus, nausea, vomiting, dry mouth, constipation, physical dependence, tolerance, and respiratory depression	-Intended dose increase -Symptomatic treatment -Sometimes discontinuation of opioid therapy
Opioid overdose/Intoxication [5]	Occurs when a person has excessive unopposed stimulation of the opiate pathway.	Flushed skin, respiratory depression, bronchoconstriction, peripheral vasodilatation, hypotension, CNS depression, miosis or mydriasis, conjunctival injection, seizures, euphoria, drowsiness, anxiety, agitation, hallucinations, depression, nightmares, gastric aperistalsis, nausea, vomiting,	-Protect Airway-Breathing-Circulation -Naloxone antidote: 0.1 mg/kg/dose (max 2 mg/dose) IV, repeat every 2–3 min if needed. (IM, SC and intranasal routes exist but more delayed onset of action) Continuous IV infusion needed sometimes. - *Isolated opioid toxicity is unlikely if no response is witnessed after administration of Naloxone.*
OIN [6]	Accumulation of active metabolites due to reduced clearance such as in liver or kidney dysfunction, elderly, dehydration… leading to CNS toxicity	Acute delirium (with agitation and hyperactivity, or hypoactivity) Confusion, myoclonus, seizures, hyperalgesia, allodynia, hallucinations	-Opioid rotation -Hydration -Symptomatic treatment of severe myoclonus with Benzodiazepines or Baclofen -*Naloxone does not treat OIN and should not be used.*
Withdrawal syndrome [7]	It is related to pathways of adenylyl cyclase superactivation-based central excitation. It occurs when a patient who is dependent on opioids suddenly reduces or stops taking opioids, or is given an opioid partial agonist like buprenorphine or antagonists like naloxone.	lacrimation or rhinorrhea, piloerection, myalgia, diarrhea, nausea/vomiting, abdominal cramping, myalgia, arthralgia, pupillary dilation, photophobia, insomnia, autonomic hyperactivity (tachypnea, hyperreflexia, tachycardia, sweating, hypertension, hyperthermia), and yawning.	-Substitution with Methadone, Buprenorphine or Buprenorphine /Naloxone, -Symptomatic treatment

*MOP* Mu opioid receptor, *DOP* Delta opioid receptor, *KOP* Kappa opioid receptor, *CNS* Central nervous system, *IV* Intravenously, *IM* Intramuscularly, *SC* Subcutaneously, *OIN* Opioid induced neurotoxicity

## Data Availability

The corresponding author should be contacted: Marianne Majdalani, mn40@aub.edu.lb.
